# The complete plastome of *Peucedanum praeruptorum* (Apiaceae)

**DOI:** 10.1080/23802359.2019.1676180

**Published:** 2019-10-16

**Authors:** Yingshuo Li, Maolin Geng, Zenglai Xu, Qiong Wang, Lingli Li, Ming Xu, Mimi Li

**Affiliations:** a Institute of Botany, Jiangsu Province and Chinese Academy of Sciences, Nanjing, PR China;; b The Jiangsu Provincial Platform for Conservation and Utilization of Agricultural Germplasm, Nanjing, PR China

**Keywords:** *Peucedanum praeruptorum* Dunn, Apiaceae, Selineae, plastome, chloroplast genome

## Abstract

*Peucedanum praeruptorum* is an important traditional herbal medicine unique to China. The complete chloroplast genome of *P. praeruptorum* was generated here using high-throughput sequencing. The plastome was 147,197 bp in size, which consisted of a pair of inverted repeats (IRs; 18,713 bp), a large single copy (LSC; 92,161 bp) and a small single copy (SSC; 17,610 bp). The GC content of the plastome was 37.6%, with 44.5%, 36.0%, and 31.1% in IRs, LSC, and SSC, respectively. A total of 128 genes were annotated, including 84 protein-coding genes, 35 tRNAs, eight rRNAs, and one pseudogene (Ψ*ycf*1). The phylogenomic analysis indicated that *P. praeruptorum* formed a monophyletic clade with *Peucedanum japonicum*.

The genus *Peucedanum* L. (tribe Selineae, Apiaceae) comprises approximately 120 species, which are widely distributed in the Old World (Sheh et al. 2005). However, the species delimitation, species relationships within the genus is still remained unclear due to morphological heterogeneous and lack of genomic information. *P. praeruptorum* Dunn is a perennial herb endemic to China. The dried roots of this plant (Peucedani Radix) are important traditional Chinese medicine called ‘Qian Hu,’ which have been used for treating anemopyretic cold and cough with accumulation of phlegm (National Pharmacopoeia Committee 2015). More than 40 species of Apiaceae were used as adulterants for Peucedani Radix (Wu et al. 2017). Here, we reported the complete plastid genome of *P. praeruptorum* to facilitate taxonomic approaches and phylogenetic inferences of *Peucedanum*, as well as herbal authentication on *P. praeruptorum.*

The fresh leaves of *P. praeruptorum* were collected from Good Agricultural Practice (GAP) base in Chun’an County, Zhejiang province, China (29°36′7.12″N, 119°2′38.94″E). The voucher specimen was deposited in the Herbarium of Institute of Botany, Jiangsu Province and Chinese Academy of Sciences (NAS, voucher No. 617194). Total genomic DNA was extracted using CTAB method (Doyle and Doyle 1987) and sequenced by Illumina Hiseq X-ten platform (San Diego, CA). Totally, 3 Gb pair-end raw reads of 150 bp were obtained for chloroplast genome assembled by NOVOPlasty version 2.7.2 (Dierckxsens et al. 2017). The complete plastome was annotated by GeSeq (Tillich et al. 2017) and adjusted by manual in Geneious version 11.1.5 (https://www.geneious.com).

The plastome sequence of *P. praeruptorum* (GenBank Accession Number: MN016968) was 147,197 bp in length. It contained two copies of inverted repeats (IRs; 18,713 bp), a large single copy (LSC; 92,161 bp), and a small single copy (17,610 bp). A LSC/IRb junction shift was detected, which was similar to the other plastomes of tribe Selineae (Samigullin et al. 2016). A total of 128 genes were identified, containing 84 protein-coding genes (PCGs), 35 transfer RNA genes (tRNA), eight ribosomal RNA genes (rRNA) and one pseudogene (Ψ*ycf*1). Among these PCGs, 10 genes (*rps*16, *rpo*C1, *rpl*16, *rpl*2, *pet*D, *pet*B, *ndh*B × 2, *ndh*A, and *atp*F) contained a single intron, two genes (*ycf*3 and *clp*P) contained two introns and one gene was trans-splicing (*rps*12 × 2). The overall GC content of *P*. *praeruptorum* plastome was 37.6%.

In order to reconstruct the phylogenetic relationships within tribe Selineae, 16 additional plastome sequences were archived from NCBI. The alignment was conducted using the MAFFT version 7.409 (Katoh and Standley 2013). And a molecular phylogenetic tree was generated based on whole plastome sequences using maximum likelihood analysis in RAxML (Stamatakis 2014). The result reveals that *P. praeruptorum* is sister to the specie of *Peucedanum japonicum* KU866530 (Figure 1).

**Figure 1.**
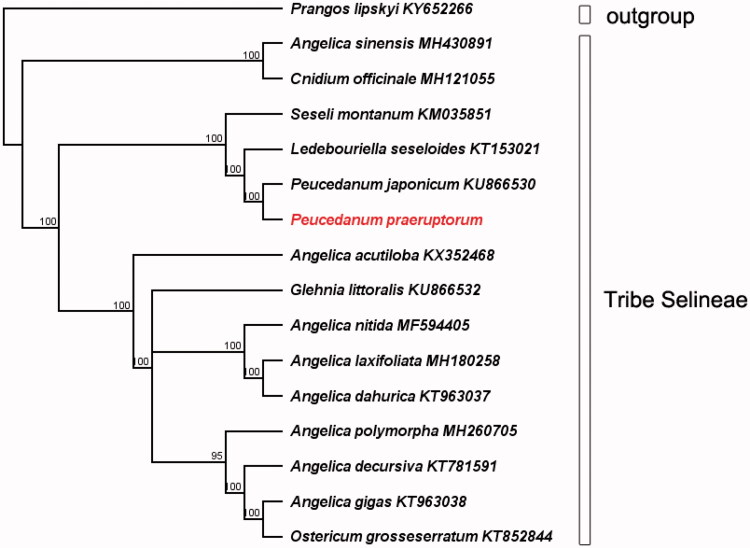
The phylogenetic tree for *Peucedanum praeruptorum* and other species in tribe Selineae base on maximum likelihood method. The bootstrap support value was labelled above each node.
